# Classification of brain lesions using a machine learning approach with cross-sectional ADC value dynamics

**DOI:** 10.1038/s41598-023-38542-7

**Published:** 2023-07-15

**Authors:** Peter Solar, Hana Valekova, Petr Marcon, Jan Mikulka, Martin Barak, Michal Hendrych, Matyas Stransky, Katerina Siruckova, Martin Kostial, Klara Holikova, Jindrich Brychta, Radim Jancalek

**Affiliations:** 1 Department of Neurosurgery, St. Anne’s University Hospital, Pekarska 53, 656 91 Brno, Czech Republic; 2 Faculty of Medicine, Masaryk University, Brno, Czech Republic; 3 Faculty of Electrical Engineering and Communication, Brno University of Technology, Technicka, 12, 616 00 Brno, Czech Republic; 4 Department of Medical Imaging, St. Anne’s University Hospital, Brno, Czech Republic; 5 First Department of Pathology, St. Anne’s University Hospital, Brno, Czech Republic

**Keywords:** Classification and taxonomy, Machine learning, CNS cancer, Brain imaging

## Abstract

Diffusion-weighted imaging (DWI) and its numerical expression via apparent diffusion coefficient (ADC) values are commonly utilized in non-invasive assessment of various brain pathologies. Although numerous studies have confirmed that ADC values could be pathognomic for various ring-enhancing lesions (RELs), their true potential is yet to be exploited in full. The article was designed to introduce an image analysis method allowing REL recognition independently of either absolute ADC values or specifically defined regions of interest within the evaluated image. For this purpose, the line of interest (LOI) was marked on each ADC map to cross all of the RELs’ compartments. Using a machine learning approach, we analyzed the LOI between two representatives of the RELs, namely, brain abscess and glioblastoma (GBM). The diagnostic ability of the selected parameters as predictors for the machine learning algorithms was assessed using two models, the k-NN model and the SVM model with a Gaussian kernel. With the k-NN machine learning method, 80% of the abscesses and 100% of the GBM were classified correctly at high accuracy. Similar results were obtained via the SVM method. The proposed assessment of the LOI offers a new approach for evaluating ADC maps obtained from different RELs and contributing to the standardization of the ADC map assessment.

## Introduction

Cerebral ring-enhancing lesions (RELs) embody a diversified group of brain pathologies with a broad differential diagnosis; in conventional magnetic resonance imaging (MRI), however, the lesions appear to be very similar^1,2^. In general, RELs can be classified into three pathological components based on MRI. The central compartment is formed by a necrotic or cystic area, surrounded by a middle compartment of a ring-like contrast enhancement due to the leaky blood–brain barrier (BBB). The peripheral part of RELs comprises perilesional edematous brain tissue characterized by T2 hyperintensity^2–4^.

The main RELs comprise, for instance, high-grade gliomas, cerebral abscesses, metastases, lymphomas, and tuberculomas.^5–8^. Early diagnosis is crucial for some brain lesions, including brain abscesses, due to the need for urgent and specific therapy. The histopathological assessment of the tissue samples requires invasive surgical intervention, and therefore increasing interest is paid to non-invasive diagnostics exploiting advanced MRI techniques. Although RELs display a similar pattern in MRI, they constitute pathologies with different biological backgrounds affecting the random motion of the water molecules in different manners^9–11^. Each pathology thus has a specific diffusion pattern that is usable for non-invasive MRI diagnostics.

Advanced MRI techniques, such as diffusion-weighted imaging (DWI), are widely employed in MRI protocols for examining brain tumors. Nonetheless, the true potential of these methods does not always materialize in practice. The quantitative expression of DWI is the apparent diffusion coefficient (ADC), which reflects the numerical value of the restriction on water molecule diffusion within the region of interest (ROI)^12^. Several factors, including increased cellularity, tissue viscosity, cellular swelling, and the consequent reduction in extracellular space, restrict the Brownian movement of water molecules “trapped” in the intracellular space, leading to decreased ADC values^13^.

The potential of ADC values in differentiating between commonly identified RELs or even glioma grading has been published^11,14–17^. Moreover, the interest in ADC values in connection with novel molecular and genetic characteristics of brain tumors, in particular, is gaining in importance^18–20^. Currently, most of the relevant research that utilizes ADC data uses ADC mean, ADC min, and ADC max in various ROIs^21–26^. However, standardization is currently lacking in the assessment of ADC maps. The differences between the methodologies applied in measuring ADC maps may be a major limitation for effective comparison of the results proposed in articles that examine the problem. The distinct features of RELs comprising 3 standard compartments give rise to uncertainty regarding the reproducibility of absolute ADC values when ROI delineation across the published studies is different. To overcome the limits of measuring ADC values, we focused on their dynamics across all the REL compartments instead of using absolute regional values. This new and more comprehensive evaluation of ADC maps could better represent the biological background of various brain pathologies. The aim of our study was to verify whether the dynamics of ADC values across the REL compartments employing cross-sectional Line of Interest (LOI) analysis can be used for the differential diagnosis of the two main pathologies, i.e., the glioblastoma (GBM) and brain abscess. In this context, a prominent objective lies in selecting suitable predictors for training machine learning algorithms to increase the probability of classifying these brain pathologies correctly.

## Materials and methods

### Population and data acquisition

This single-center retrospective study was conducted at the Neurosurgical Department, St. Anne’s University Hospital, Brno, Czech Republic. In total, we enrolled 40 patients operated on for REL with subsequent histopathological verification between January 2017 and December 2022; of these subjects, 26 individuals (11 males, 15 females) were diagnosed with GBM (according to 2016 WHO classification) and 14 (8 males, 6 females) with brain abscesses. Each patient underwent an MRI examination (GE Discovery 750w, 3 T) at the Department of Diagnostic Imaging, St. Anne’s University Hospital, one day before surgery. The MRI protocol included 3D T2-weighted images, axial T2 FLAIR, 3D T1-weighted images, 3D contrast-enhanced T1-weighted images, and diffusion-weighted imaging^27^. DWI parameters were TR/TE = 6000/100 ms, slice thickness 5 mm, gap 1.5 mm, FOV 240 × 240 mm, matrix 256. The diffusion changes were analyzed via vendor software-based ADC calculation from images acquired using three diffusion gradients with two b values (0 and 1000 s/mm^2^). The MRI data of the patients were collected in the DICOM format, anonymized, and analyzed retrospectively.

### Data processing

**Figure 1:**
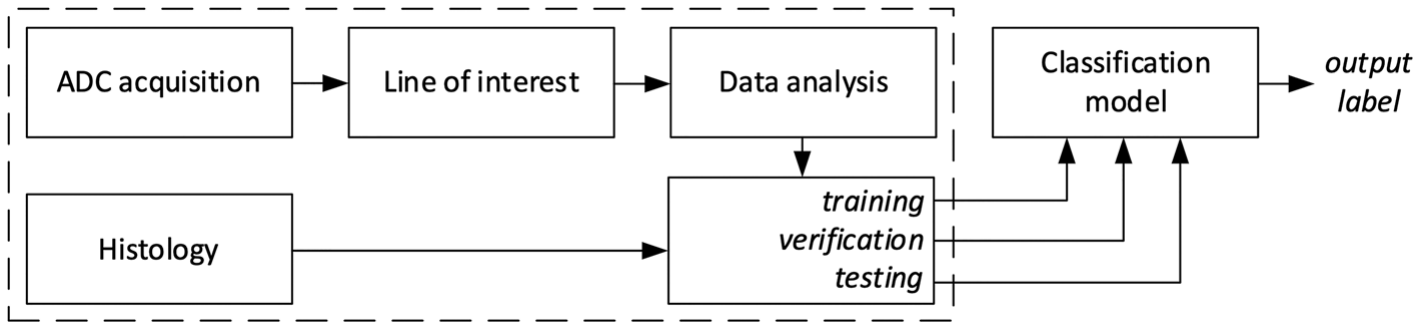
Block diagram of pathological tissue processing and classification. The output of the MRI is a set of scans that can be expressed as a matrix of ${\mathbf{ADC}}$ values with spatial coordinates $ADC(x,y,z)$. A user-defined region of interest can be described by a line equation in the space ${\mathbf{R}}_{{{\text{OI}}}} = {\mathbf{A}} + t \cdot {\mathbf{u}}$, where ${\mathbf{R}}_{{{\text{OI}}}}$ is a vector of coordinates of points on the line, ${\mathbf{A}}$ is a point in the space of scanned ADC values, and ${\mathbf{u}}$ is a direction vector. The output of the ADC acquisition block is the vector of ADC values lying on the line ${\mathbf{R}}_{{{\text{OI}}}}$, i.e., ${\mathbf{ADC}} \cap {\mathbf{R}}_{{{\text{OI}}}}$.

An advanced computer analysis was carried out at the Department of Theoretical and Experimental Electrical Engineering, Faculty of Electrical Engineering and Communication, Brno University of Technology, Brno, Czech Republic. The processing chain is shown in Fig. 1. The information from the scanned ADC images was compressed by acquiring user-selected values under a linear region with 1-pixel width. Together with the histological result, the vector of the compressed ADC values formed a set to train the classification models. The output of the classifier is the predicted label of the classified lesion.

### Cross-sectional ADC analysis of ring-enhancing lesions

To classify the brain pathologies, we created a program in Matlab R2020b that allowed us to identify the data acquired within the line segment inserted into the image slice and to analyze the selected data; such a procedure then facilitated classifying the pathological tissue.

**Figure 2:**
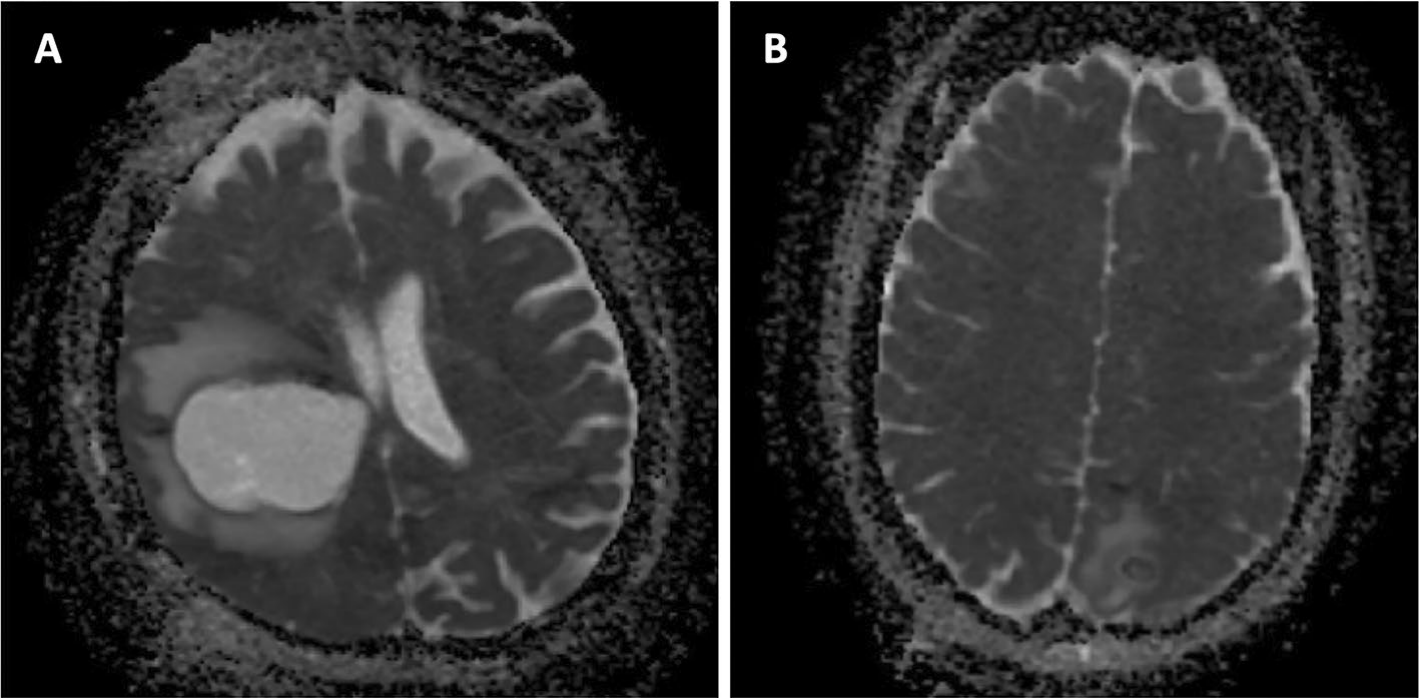
The representative images of the examined pathologies on ADC map calculated from DWI with b value 0—(**A**) glioblastoma, (**B**) abscess.

A single representative slice of the ADC map showing the REL in the longest diameter as well as containing the characteristic perilesional edema was chosen by a skilled clinician for every patient on the diagnostic MRI and loaded into the program to perform the analysis (Fig. 2). For this purpose, five linear lines of interest (LOI) were placed into a single representative slice of the ADC map. The LOI function was programmed in Matlab to approximate the ADC values between the selected points linearly. The ADC values were then saved as a vector.

In total, 200 cross-sectional lines were analyzed, from which 150 lines from 30 different subjects (75% of the data) were used to train the classification algorithms, and 50 lines from another 10 subjects (25% of the data) were used for the hypothesis testing. Each radially oriented LOI crossed all compartments of the REL, beginning in an apparently healthy-looking tissue adjacent to perifocal edema with the ADC values close to normal, around 700–750 mm^2^/s for brain parenchyma^28–30^ going across the region of the perilesional brain parenchyma that exhibits contrast enhancement and ending in the central core of the REL.

Areas with a specific deviation of the ADC values, extremely high in the brain ventricle and brain cisterns while extremely low in the bone and calcifications were avoided, as were analysis of 2 contralaterally oriented LOI across the REL.

After that, the specific points were marked on the LOI by a skilled radiologist: A) the most distant border of the markable perilesional edema; B) the outer border of the most intense ADC hyperintensity caused by the perilesional edema; C) the inner border of the ADC hyperintensity area close to the tumor border, right before the ADC starts to decrease; D) the most intense ADC hypointensity representing the REL border with the most intense restriction of a diffusion; E) the border of the content of the REL, usually characterized by a homogenous area of a higher ADC value; F) the end of the LOI in the center of the REL.

The dynamic changes in the ADC values along the cross-sectional LOI were marked as a curve in the graph of the ADC value [mm^2^/s] plotted on the y-axis. The points of interest outlined above were defined on this curve, as follows: (A) the beginning of the elevation of the ADC values at the most distant part of the perilesional brain edema; (B) the point of stabilization of the elevated ADC values, the beginning of a plateau on the curve of ADC values in the perilesional brain edema; (C) the point where the ADC values start to decrease to the lower values around the enhancing ring; (D) the lowest value between the perilesional brain edema and the enhancing ring; (E) the point of stabilization of the ADC values in the REL core and (F) the final point on the curve extending to the center of the RELs.

Using these points, the curve was then divided into characteristic segments: (A-B) gradient 1 (onset of the perilesional brain edema); (B-C) plateau 1 (the perilesional brain edema, including the fluctuation of the elevated ADC values); (C-D) gradient 2 (the transition between the perilesional brain edema and the enhancing ring); (D-E) gradient 3 (the transition between the enhancing ring and the REL core); (E–F) plateau 2 (the REL core).

The following general equation was used to calculate the gradient $\nabla f$ of the function $f\left(x\right),$ which describes the intensity of the ADC map under the LOI:

1 $$\nabla {f}_{{x}_{0}}\approx \frac{f\left(x\right)-f\left({x}_{0}\right)}{x-{x}_{0}},$$

where *x* and *x*_0_ are the *x*-coordinates of the selected points on the *x*-axis; *f*(*x*) and *f*(*x*_0_) denote their functional values.

### Data analysis

**Figure 3:**
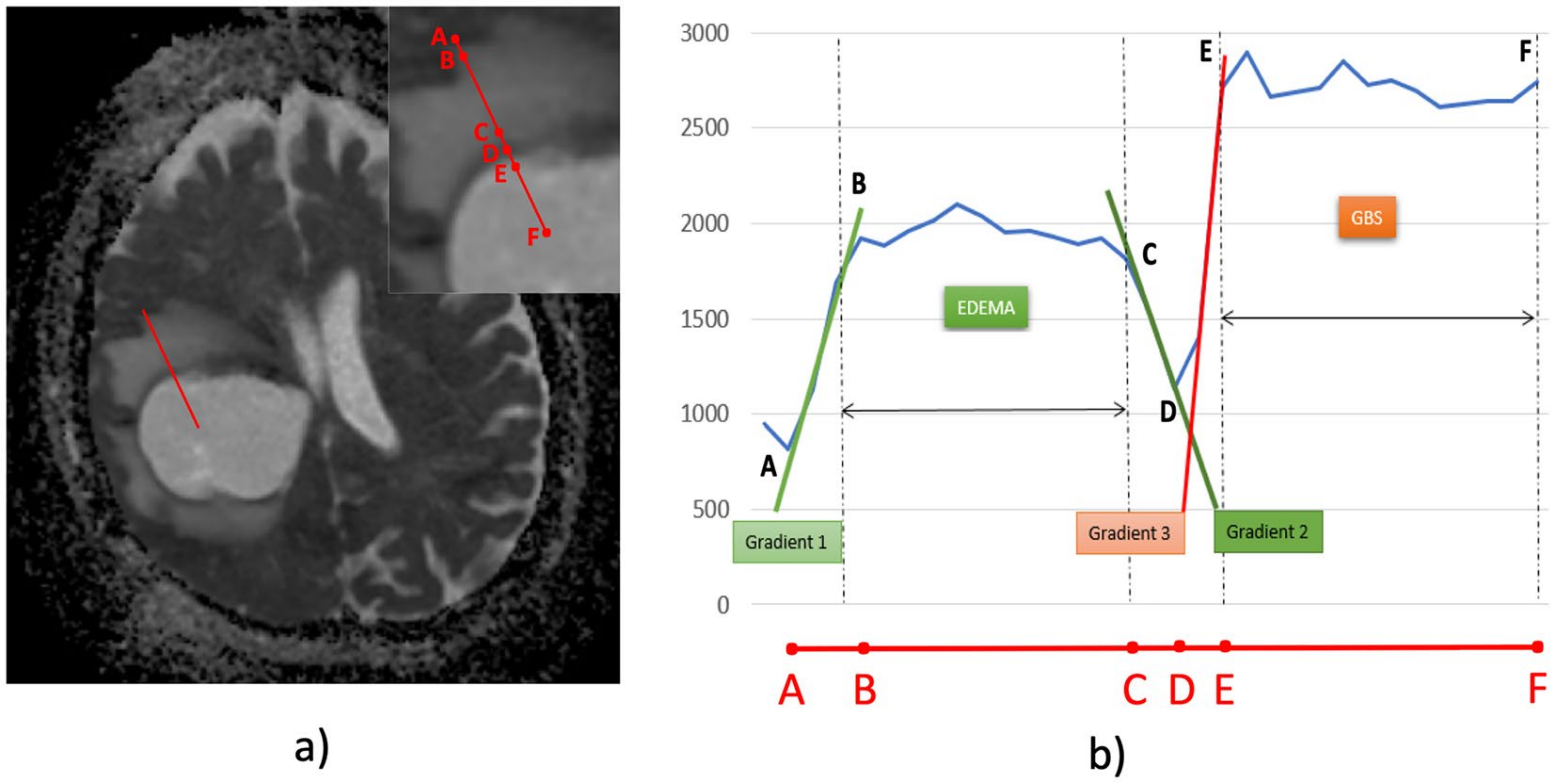
(**a**) Image slice and a line of interest (LOI) running from the periphery to the core of the ring-enhancing lesion. Inset shows a higher magnification of the region marked by the LOI, including its division using points A–F. (**b**) Graph showing the selected points from the LOI.

The analysis of the data defined by the LOI was carried out in order to select the predictors for the machine learning algorithms. Figure 3b shows the waveform of the values represented by the red LOI (Fig. 3a). Based on the data analysis, the following predictors were chosen (from left to right): the gradient of the leading edge of the onset of the perilesional brain edema (gradient 1), $\nabla ADC_{{{\text{E}}_{rise} }}$; the median ADC values (plateau 1), $\overline{{ADC_{{\text{E}}} }}$; the standard deviation of the ADC values, $\sigma_{{ADC_{{\text{E}}} }}$(plateau 1); the transition between the perilesional brain edema and the enhancing ring (gradient 2), $\nabla ADC_{{{\text{E}}_{fall} }}$; the transition between the enhancing ring and the REL core (gradient 3), $\nabla ADC_{{{\text{T}}_{rise} }}$; the median ADC values (plateau 2), $\overline{{ADC_{{\text{T}}} }}$; the standard deviation of the ADC values, $\sigma_{{ADC_{{\text{T}}} }}$ (plateau 2). To discriminate between the glioblastoma and abscess, a set of boxplots was created.

The data used for the box plots were also analyzed using statistical tests (the non-parametric Wilcoxon test and the Kruskal–Wallis test). A test of the normality of the data was employed first, followed by the appropriate test to determine the statistical significance of the data under study.

### Data classification

The input of the classifier can be defined as a vector ${\mathbf{fv}}$ containing the features and, in the training phase, also the result of a histological examination that corresponds to the actual classification of the pathology:

2 $${\mathbf{fv = }}\left( {\nabla ADC_{{E_{rise} }} ,\overline{ADC}_{E} ,\sigma_{{ADC_{E} }} ,med_{{ADC_{E} }} ,\nabla ADC_{{E_{fall} }} ,\nabla{ADC}_{{T_{rise} }} ,\overline{ADC}_{T} ,\sigma_{{ADC_{T} }} ,med_{{ADC_{T} }} } \right)$$

To classify the data, we used two models: the k-NN model and the SVM model with a Gaussian kernel. The relevance of the models was tested via fivefold cross-validation. Both models classified the lesion type based on the input vector containing the nine characteristics mentioned above.

### k-NN model

The k-Nearest Neighbors algorithm (k-NN) is a method of supervised machine learning that can identify patterns via classifying features represented by multidimensional vectors into two or more classes. At the training stage, the training set is preprocessed so that all the symptoms have the mean value of 0 and variance of 1; the results are then placed, by each feature of the training set, in the N-dimensional space. During the classification stage, the feature in question is placed in the same space, and the number k of its nearest neighbors is identified; the feature in question is then classified in the same class as most of its nearest neighbors.

### SVM model

The Support Vector Machine algorithm (SVM)^31^ is trained by maximizing the distance between the marginal samples (also referred to as support vectors) and a discriminative hyperplane by maximizing the *f* in the equation:

3 $$f(\alpha_{1} , \ldots ,\alpha_{n} ) = \sum {\alpha_{i} - \frac{1}{2}\sum\limits_{i} {\sum\limits_{j} {\alpha_{i} \alpha_{j} y_{i} y_{j} \vec{x}_{i} \cdot \vec{x}_{j} } } }$$

where *y* is defined as + 1 for a class A sample and –1 for a class B sample, *α* is the Lagrangian multiplier, and $\vec{x}$ is the feature vector of the individual sample. On real data, this is often too strict because of noisy samples that might cross their class boundary. This is solved using a combination of techniques known as the kernel trick and soft margining^32,33^.

### Classification performance

**Figure 4:**
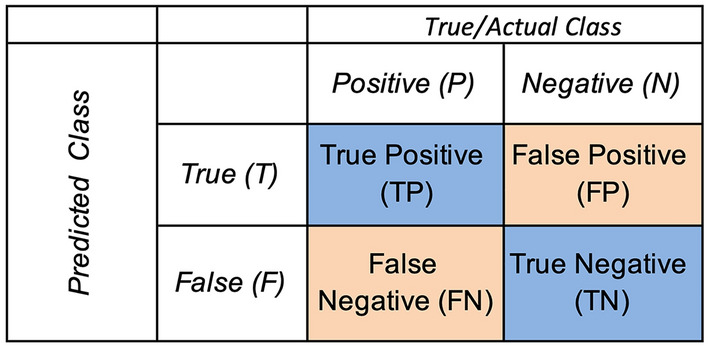
An example of a confusion matrix.

A confusion matrix is used to evaluate the results of the machine learning algorithms. There are two classes, *P* and *N*. The output of the predicted class is true or false. The blue diagonal represents the correct predictions, and the orange diagonal indicates the incorrect predictions (Fig. 4). If the sample is positive and is classified as such, i.e., a correctly classified positive sample, it counts as a true positive (TP); if it is classified as negative, it is considered a false negative (FN) or Type II error. If the sample is negative and classified as negative, it is regarded as true negative (TN); if it is classified as positive, it counts as false positive (FP)^34^.

One of the most commonly used classification performance measures is Accuracy (Acc). It is defined as a ratio between the correctly classified samples and the total number of samples^35^:

4 $$Acc=\frac{TP+TN}{TP+TN+FP+FN}.$$

Another data evaluation metric comprises sensitivity and specificity. The sensitivity – or, by another definition, the true positive rate (*TPR*) or recall—of a classifier relates the positive correctly classified samples to the total number of positive samples, and it is expressed according to Eq. (4)^35^:

5 $$TPR=\frac{TP}{TP+FN}.$$

The next evaluated quantity was the false negative rate (FNR), namely, the proportion of incorrectly classified positive samples. Thus, the FNR complements the sensitivity measure and is defined as

6 $$FNR=\frac{FN}{FN+TP}=1-TPR.$$

To suppress the effect of over- and underfitting the models, we relied especially on the cross-validation method and the early-stopping method, the latter allowing us to stop whenever a small change occurred in the model performance on the validation dataset. Furthermore, the accuracies during the training, validation, and testing were compared.

### Ethical approval

This retrospective study was approved by the Ethics Committee of St. Anne’s University Hospital Brno (registry no. EK-FNUSA-06/2023). Written informed consent was not required for this retrospective study because all patient identifying information was removed from the study data; the requirement of consent was waived by the Ethics Committee of St. Anne’s University Hospital Brno. All methods were performed in accordance with institutional and national ethical standards and with the 1964 Helsinki Declaration and its later amendments or comparable ethical standards.

## Results

### Cross-sectional analysis of the ADC maps

**Figure 5:**
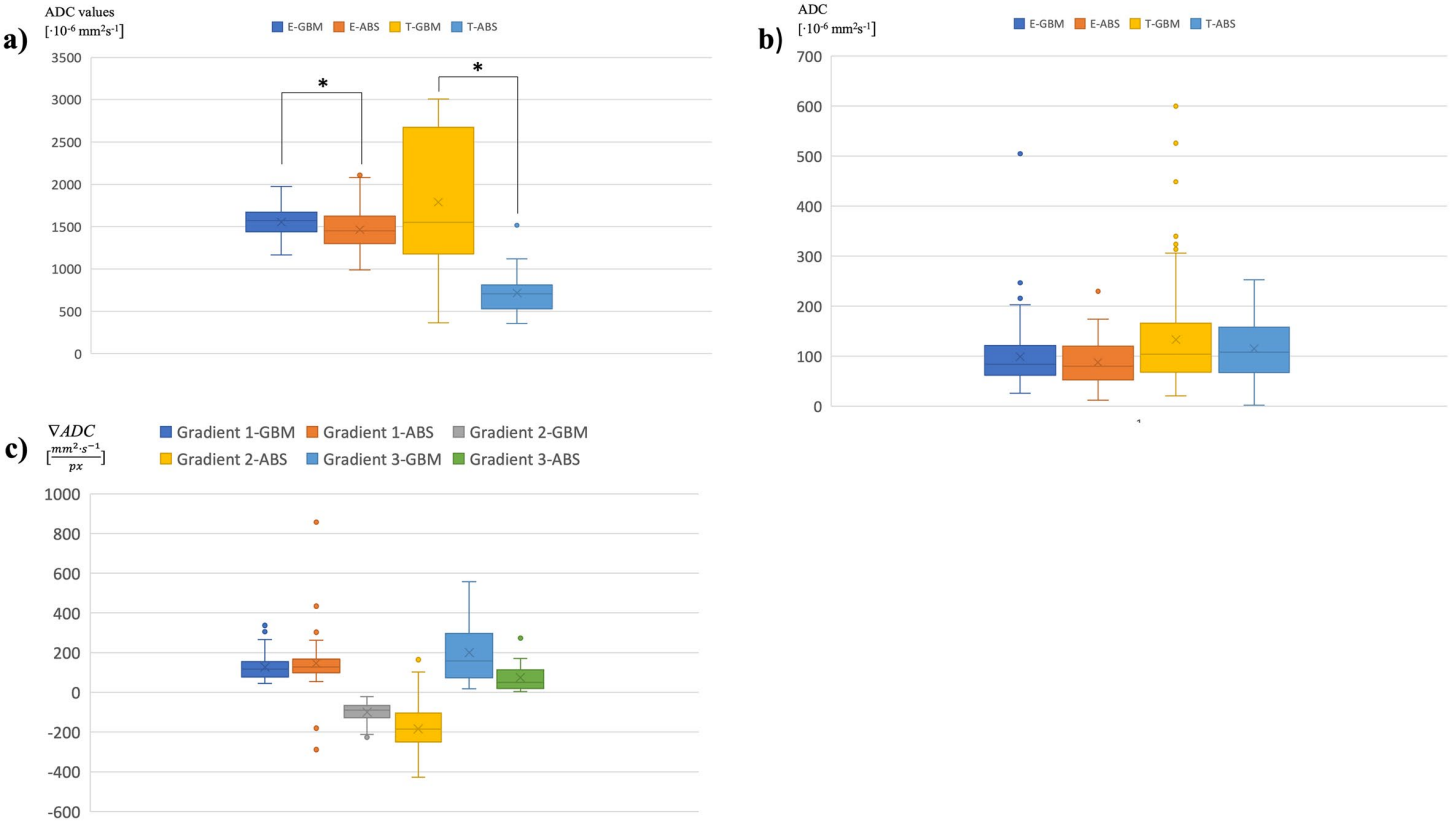
(**a**) The box plot shows the median of the ADC values obtained from the plateau (plateau 1) of perilesional brain edema in GBM (E-GBM) and brain abscess (E-ABS) and the median of ADC values from the core of these RELs (plateau 2), both the GBM (T-GBM) and brain abscess (T-ABS). * indicates a significant difference compared to the median ADC obtained from perilesional brain edema and the core of enhancing lesions between GBM and brain abscess, respectively (p < 0.05). (**b**) The box plot shows statistically non-significant differences between standard deviations of ADC values obtained from perilesional brain edema (E-GBM: glioblastoma, E-ABS: abscess) as well as the enhancing core (T-GBM: glioblastoma, T-ABS: abscess) of GBM and brain abscess, respectively. (**c**) The box plot shows statistically non-significant differences between individual gradients (gradients 1, 2, and 3) of ADC values obtained from the appropriate segment on the LOI (GBM: glioblastoma; ABS: abscess).

The relevant boxplot (Fig. 5a) shows, on the one hand, the median of the ADC values obtained from the perilesional brain edema (plateau 1; $med_{{ADC_{{\text{E}}} }}$; E-GBM: glioblastoma; E-ABS: abscess) and, on the other, the median of the ADC values for the core of the GBM and the brain abscess (plateau 2; $med_{{ADC_{{\text{T}}} }}$; T-GBM: glioblastoma; T-ABS—abscess). The median ADC values were calculated from the respective LOI data related to the pathologies in question.

The data from each boxplot (Fig. 5a–c), taken separately, do not have a normal distribution. Using the non-parametric Wilcoxon statistical test, we found that there was a statistically significant difference between the median ADC values obtained from the perilesional brain edema (plateau 1; p < 0.05) in the GBM and those from brain abscess. Similarly, there was a statistically significant difference (p < 0.05) in the median ADC values obtained from the plateau 2 region in the core of the two pathologies.

Comparisons of the standard deviations of the ADC values in the perilesional brain edema (plateau 1; $\sigma_{{ADC_{{\text{E}}} }}$; E-GBM: glioblastoma; E-ABS: abscess) and the standard deviations of the ADC values in the core of the enhancing lesions (plateau 2; $\sigma_{{ADC_{{\text{T}}} }}$; T-GBM: glioblastoma; T-ABS—abscess) are shown in Fig. 5b. The standard deviations were calculated from the respective LOIs data related to the pathologies in question.

The standard deviation values derived from the GBM and the brain abscess did not have a normal (or Gaussian) distribution. Using the Wilcoxon test, we found that the standard deviation of the ADC values obtained from the perilesional brain edema (plateau 1) in the GBM was not significantly different compared to the standard deviation of the ADC values in the brain abscess. Similarly, the differences in the standard deviation of the ADC values between the enhancing cores (plateau 2) of both RELs were not statistically significant.

The analysis of all the edges processed is shown in Fig. 5c: the gradient of the leading edge of the onset of the perilesional brain edema (gradient 1; $\nabla ADC_{{{\text{E}}_{rise} }}$), the gradient of the decreasing edge of the transition between the perilesional brain edema and the enhancing ring (gradient 2; $\nabla ADC_{{{\text{E}}_{fall} }}$), and the gradient of the leading edge of the transition between the enhancing ring and the enhancing core (gradient 3; $\nabla ADC_{{{\text{T}}_{rise} }}$); the gradients are displayed in detail in Fig. 3

The data representing the individual gradients did not show a normal distribution either. The non-parametric Kruskal–Wallis test exhibited no statistically significant differences between the data obtained from the individual gradients (gradients 1, 2, and 3) for the GBM and the brain abscess.

### Machine learning

**Figure 6:**
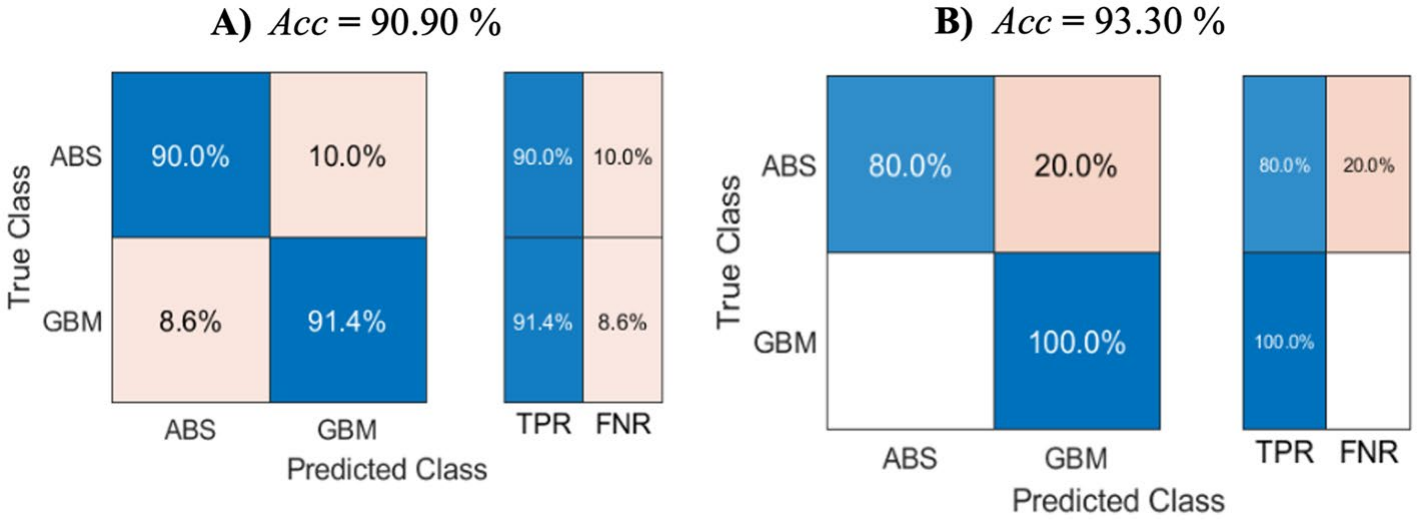
Confusion matrix, True positive rate (TPR), and False negative rate (FNR) for the training (**a**) and testing (**b**) stages of the k-NN model.

**Figure 7:**
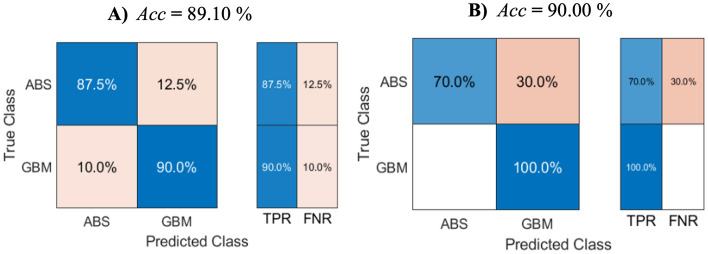
Confusion matrix, True positive rate (TPR), and False negative rate (FNR) for the training (**a**) and testing (**b**) stages of the SVM model.

The data described by the box plots were used for the machine learning method. A confusion matrix and the classification performance parameters to classify the training data via the k-NN model (Fig. 6) and the SVM model (Fig. 7), based on the 7 predictors (Table 1) representing the data related to the perilesional edema, enhancing core of the RELs, and edges between the transition zones according to the LOIs.

**Table 1:** List of predictors used for machine learning.

Predictors for machine learning	Line of interest
Onset of perilesional brain edema	A–B (gradient 1)
Median of ADC values in perilesional brain edema	B–C (plateau 1)
Transition zone between perilesional brain edema and enhancing ring	C–D (gradient 2)
Transition zone between enhancing ring and enhancing core	D–E (gradient 3)
Median of ADC values in center of enhancing core	E–F (plateau 2)
Standard deviation of ADC values obtained from perilesional brain edema	B–C (plateau 1)
Standard deviation of ADC values obtained from enhancing core	E–F (plateau 2)

However, the k-NN method performed better, with a testing accuracy of 90% in the abscess classification; the GBM, however, exhibited an almost flawless performance.

## Discussion

The conventional MRI remains the standard method of modern neuroimaging, providing general information about the location and extent of various brain pathologies. Despite these state-of-the-art imaging methods, differentiating some RELs remains a challenge. In addition to the conventional MRI approaches, such as T1 and T2 weighted images, advanced methods like DWI and its numerical expression by ADC maps are widely used. The ADC values reflect the restriction of water molecule diffusion and thus better characterize the biological background of the various RELs^36^. However, the ADC map acquisition and assessment methodology has not been standardized and lacks uniformity, mainly because of the different types of REL segmentation. Comparing the ADC values between the studies is severely limited by these issues, together with the inter-individual as well as intra-individual variations of the different RELs. We present a novel method for measuring the ADC values, using a cross-sectional analysis of the ADC value dynamics.

The standard method to measure the ADC values in the different RELs is based on compartmentalization according to T1 weighted images on MRI. Generally, studies using The ADC values to distinguish similar RELs focused on three different compartments: the core, the contrast-enhanced ring, and the perilesional zone corresponding to the brain edema^3,37,38^.

The REL core usually represents the region of necrosis marked by high metabolic requirements, strong hypoxia, or reaction to internal or external harming agents (cytokines, reactive oxygen species, microbial toxins, etc.). Due to the action of these factors, cellular components such as membranes, organelles, and proteins become fragmented and dissolve, and the viscosity of the contents of the central region is thus variable. In many RELs, the core is formed by exudation or secretion from the pathological tissue. In these cases, the central part of the RELs has a more liquid character, low cellularity, and low metabolic activity; thus, there is no contrast enhancement in the MRI. These alterations in the core of the RELs are associated with the viscosity, which negatively affects the diffusibility of the water molecules, resulting in lower ADC values. This is a dominant hallmark for brain abscesses, where the central cavity contains dead neutrophils, bacteria (or fungi/parasites), and necrotic cellular detritus leading to high viscosity and low ADC values. Conversely, an increased diffusibility and the consequent higher ADC values are more often found in glioblastoma^2,39,40^. Due to the heterogeneity of the RELs, the core may not provide a typical image on the ADC maps.

The REL core is surrounded by a contrast-enhanced ring corresponding to a higher cell density, typical of brain tumors. This layer is usually characterized by pronounced destruction of the BBB, higher neovascularization, and increased vascularity with tight extracellular spaces, resulting in diffusion restriction and lower ADC values^41–43^. However, the biological background of the enhancing ring is different in those RELs that were caused by infectious etiology. In the case of a brain abscess, the enhancing ring is formed by a thick fibrous capsule containing fibroblasts, newly formed vessels, collagen fibers, and immune cells or Langhans giant cells in a tuberculoma^44^.

The vasogenic edema around RELs forms the background of the peripheral compartment. Vasogenic brain edema is characterized by alterations in the blood–brain barrier (BBB) as a reaction of the surrounding brain tissue to the pathology, resulting in fluid leakage from the vessels to the brain parenchyma. In addition to the elevated BBB permeability, perilesional edemas are supported by forming new and aberrant brain vessels around some RELs. These changes increase the extracellular space and lead to a higher ADC^45,46^.

The ADC map measurements are currently based on the standard radiological concept of dividing RELs into the 3 compartments mentioned above. However, the histological findings show that the boundaries between the compartments (the core, enhancing ring, and perilesional edema) are not sharply delineated. Due to this fact, we can assume that a standard measurement of the ADC values in selected LOI is accompanied by a loss of some valuable information on the transition zone between the compartments. Thus, our study defines three more bridging compartments where the ADC values change dynamically: a) the transition between the core and the enhancing ring, b) the transition between the ring and the perilesional brain edema, and c) the transition between the perilesional brain edema and the normal-like brain tissue. To verify the usability of these additional zones and the impact on the differential diagnosis of the RELs, we focused on an LOI crossing all standard compartments and transition zones in the major RELs, i.e., the GBM and the brain abscess.

The LOI periphery is characterized by a transition between the healthy brain tissue and the perilesional brain edema. Using our method of cross-sectional LOI analysis in the centripetal direction, we established that the ADC gradient at the onset of the perilesional brain edema showed no differences between the GBM and the brain abscess (Fig. 5c). This finding is in accordance with those by Toh et al., who presented no significant differences in the ADC values in the distal zone of the perilesional brain edema between both pathologies^11^. The result proves the hypothesis that the adjacent brain edema compartment represents a common reaction of the brain tissue to various pathologies caused by the structural disintegration of the BBB endothelial cells. However, the perilesional edema in the GBM is affected by the glioma cells scattered around the tumor mass^45^. This biological background in the GBM was probably reflected in the cross-sectional LOI analysis across the perilesional brain edema compartment that showed a significant increase in the median ADC values in the GBM compared to the brain abscess (Fig. 5a).

Approaching the enhancement ring, the brain edema becomes more infiltrated by cells corresponding to the pathologies; therefore, the transition zone between the perilesional edema and the enhancing ring is characterized by a decline in the ADC values. Although our result supports the finding by Toh et al., who showed different ADC values in the immediate zone of the perilesional brain edema between the GBM and the brain abscess, the authors did not account for the dynamics of the ADC values through the transmission compartment^11^. Compared to previous studies, the dynamics of the ADC values in this transition compartment provide valuable information which is not obvious in standard ADC map evaluation. Additionally, the gradient between the perilesional brain edema and the enhancing ring was lower in the brain abscess, thus suggesting that the gradient of the LOI in the brain abscess was higher than in the GBM (Fig. 5c).

This pattern might be at least partially explained by the massive infiltration of the glioma cells into the brain edema in the proximity of the enhancing ring, resulting in lower ADC values compared to the brain abscess. In addition to the infiltrative glioma cells, vascular proliferation may also play a significant role, similarly to the tumor-associated immune cells present in the border zone between the edema and the enhancing ring in the GBM. These changes lead to a restriction in the water diffusion, thus reducing the ADC values. Contrariwise, a brain abscess is a circumscribed form of brain inflammation characterized by limited cellular infiltration into the perilesional brain edema; therefore, the increase in the ADC values is more rapid. Also, the products of the inflammatory cells, as well as bacterial toxins, contribute to increased vascular permeability and increased ADC values.

The compartment of the enhancing ring is too thin for a broad analysis of the ADC values due to the low resolution of the ADC maps. Moreover, this compartment has been partially evaluated by adding transition compartments directly adjacent to the enhancing ring; thus, we did not compare this compartment in our analyses.

The central transition zone is the intermediate area between the enhancing ring and the inner core. Using our method of cross-sectional LOI analysis, we found a lower but not significant slope of the ADC gradient in the GBM when compared to the brain abscess (Fig. 5c). This may be due to the high proliferation rate in the intermediate area of the GBM, leading to high cellularity^43^.

The central part of the REL is the most heterogeneous region reflecting the pathology background mentioned above. In general, higher ADC values in the GBM core correspond to the extensive necrotic area with high water diffusion. On the other hand, an elevated viscosity in the core of the brain abscess leads to restricted diffusion, resulting in low ADC values^2,39,40^. This is in accordance with the result of our cross-sectional LOI analysis, which exposed higher median ADC values in the GBM core compared to the brain abscess (Fig. 5a). However, in some cases, higher ADC values typical of the GBM were also found in brain abscesses^11^. Conversely, lower ADC values mimicking brain abscesses were also found in the GBM^11,47^.

We found several differences in the assessed parameters using the cross-sectional LOI analysis and a subsequent comparison between the GBM and the brain abscess targeting the characteristics of each not only main but also transitional compartment (Fig. 5a–c). However, using statistical tests, the differentiation of the GBM from the brain abscess was not very accurate when comparing the individual parameters obtained by analyzing the ADC values in the ROI cross-section. We found that the data obtained from the LOI did not have a normal distribution; therefore, to increase the ability to distinguish the GBM from the brain abscess, we included all of the parameters in a multiparametric evaluation using machine learning algorithms. In Table 1, we can observe an overview of all the predictors derived from the LOI and subsequently used in machine learning. Our methods were able to distinguish the two representative RELs, meaning the GBM and the brain abscess, at high accuracy (Figs. 6 and 7). To classify the studied pathologies, we used two machine learning methods, namely, the k-NN and the SVM. Utilizing the test data, we achieved very good results for both methods. However, the k-NN method performed better, with 80.0% of the abscesses classified correctly during the testing; the GBM delivered an accuracy of 93.3%. Using the SVM method, a correct prediction was achieved in 70.0% of the abscesses, and 90.0% of the glioblastomas. However, in future research, we still plan to use the two methods, albeit complemented with other convenient approaches and datasets: Combining such tools and options appears to have the potential of delivering very promising results.

Unlike the previous methods, the proposed methodology for discriminating between the two RELs with different pathological backgrounds joins together the advantage of very fast diagnosis with simplicity and uniformity in the data extraction, processing, and interpretation. Moreover, in contrast to the commonly published approaches, there is no need for multidimensional image analysis, spatial modeling, volumetry, or other complex ADC image processing operations.

Despite the promising results, our study has several limitations. To define the brain tissue structures that affect the ADC value, we have to describe the size of a pixel on the ADC maps. We analyzed ADC maps with the pixel size of 0.8 × 0.8 mm and slice thickness of 5 mm; this means that the ADC value for a voxel was calculated from the volume of 3.2 mm^3^. However, the biological basis of an area of this size can be heterogeneous, containing different numbers and types of cells, vessels, extracellular matrices, and other elements. Nonetheless, one pixel could comprise multiple GBM or brain abscess zones, including transition ones.

Another limitation is the small number of patients, partially compensated for by the higher volume of LOIs acquired from the GBM and the brain abscess. Moreover, the number of patients varied between the individual groups. In general, brain abscesses are less common than GBMs; thus, there were twice as many patients with a GBM than with a brain abscess.

## Conclusions

Our study proposes a new approach for evaluating ADC maps obtained from different RELs. Along with an assessment of the ADC value dynamics in the individual REL compartments, including transitional ones, we were able to distinguish GBMs from brain abscesses with high accuracy. Moreover, the proposed method can contribute to standardization in ADC map evaluation. Considering the promising results of our procedures for evaluating the ADC values in different compartments of RELs, the effectiveness of the research outcomes is presently verifiable in prospective clinical trials.

## Data Availability

The datasets analyzed during the current study are available from the corresponding author upon reasonable request.
